# Echocardiographic diagnoses in HIV-infected patients presenting with cardiac symptoms at Muhimbili National Hospital in Dar es Salaam, Tanzania

**DOI:** 10.5830/CVJA-2011-060

**Published:** 2012-03

**Authors:** Pilly Chillo, Muhammad Bakari, Johnson Lwakatare

**Affiliations:** Muhimbili National Hospital and Muhimbili University of Health and Allied Sciences, Dar es Salaam, Tanzania; Muhimbili National Hospital and Muhimbili University of Health and Allied Sciences, Dar es Salaam, Tanzania; Muhimbili National Hospital and Muhimbili University of Health and Allied Sciences, Dar es Salaam, Tanzania

**Keywords:** HIV and cardiac symptoms, echocardiographic diagnoses in HIV

## Abstract

**Objective:**

To determine the pattern of echocardiographic diagnoses in HIV-infected patients presenting with cardiac symptoms at Muhimbili National Hospital in Dar es Salaam, Tanzania.

**Methods:**

Patients known to be HIV positive and with cardiac complaints were prospectively recruited from the Hospital’s care and treatment centre as well as from the medical wards. Clinical assessment, laboratory tests and echocardiography were performed.

**Results:**

A total of 102 patients were recruited from September 2009 to April 2010. The patients’ mean age was 42.4 years and 68.6% were women. The most common diagnosis was pericardial effusion present in 41.2% of the patients. The effusion was large in 5.9% and small in 35.3% of the patients. Hypertensive heart disease was diagnosed in 34.3%, while pulmonary hypertension and dilated cardiomyopathy were present in 12.7 and 9.8%, respectively.

**Conclusion:**

Cardiac abnormalities are common in HIV-infected patients, particularly when they present with symptoms.

## Abstract

Infection with the human immunodeficiency virus (HIV) is known to cause various cardiac abnormalities as a consequence of the direct viral effect on cardiac tissue^1^ or opportunistic diseases.^2^ Furthermore, the improved survival and ageing of HIV-infected patients following the use of highly active antiretroviral therapy (HAART) has been associated with the presentation of chronic late complications, including heart diseases.^3^

Studies from the pre-HAART era documented a predominance of infectious conditions and cardiomyopathies as the main aspects of cardiac involvement in HIV-infected patients.^4^-^6^ However, recent publications, mainly from Europe and North America, have reported an increase in prevalence of cardiovascular risk factors in HIV-infected patients on HAART, ranging from increased prevalence of hypertension and deranged cholesterol profiles to impaired glucose tolerance.^7^,^8^ This increased prevalence of traditional cardiovascular risk factors among HIV-infected patients may have an impact on the pattern of cardiac diseases with which these patients will present.

In sub-Saharan Africa, several studies have been carried out to determine the prevalence of cardiac diseases among HIV-infected patients. Twagirumukiza *et al.* found the prevalence of dilated cardiomyopathy to be 17.7% in a fairly large sample of 416 HIV-infected patients.^9^ In a review by Magula *et al.*, cardiomyopathy and pericardial diseases were reported as the commonest cardiac involvements among HIV-infected patients in Africa.^10^

In Tanzania, previous studies on cardiac involvement in HIV-infected patients were carried out in the pre-HAART era.^4^,^11^ Data are lacking on the pattern of cardiac involvement in HIV-infected patients in this new era of HAART. The aim of the present study was therefore to determine the pattern of cardiac abnormalities among HIV-infected patients presenting with cardiac symptoms at Muhimbili National Hospital in Dar es Salaam, which is the only tertiary referral health facility in Tanzania.

## Methods

This was a cross-sectional, hospital-based study conducted at Muhimbili National Hospital between September 2009 and April 2010. Patients were prospectively recruited from the Hospital’s outpatient care and treatment centre (CTC), as well as from the medical wards.

Patients known to be HIV positive and presenting with any of the following complaints: palpitations, shortness of breath (SOB), orthopnoea, paroxysmal nocturnal dyspnoea (PND), oedema of the lower limbs, cough (thought to be of cardiac origin) and non-pleuritic chest pain, were consecutively recruited. Patients were excluded if they were under 18 years old and if they did not consent to participate.

All participating patients signed a written consent form. The study received ethical approval from the local institutional ethical review board, referred to as the MUHAS’s Senate Research and Publications Committee.

In order to detect a 25% difference between patients with a CD_4_^+^ cell count < 100 cells/μl and those with a cell count ≥ 100 cells/μl at a 5% significance level and 80% power, a minimum of 90 patients was required.^12^ This was based on previous findings that cardiac diseases occurred in 31% of patients with a CD_4_^+^ cell count < 100 and in 6% in those whose CD_4_^+^ cell count was ≥ 100 cells/μl.^13^

A structured questionnaire was used to collect socio-demographic characteristics, other cardiovascular risk factors, and history of antiretroviral drugs used. Height and weight measurements were recorded and were used to determine body mass index (BMI). Blood pressure was taken using a mercury sphygmomanometer; a set of three readings, and the average of the last two readings was taken as the patient’s office blood pressure. Hypertension was defined as blood pressure ≥ 140/90 mmHg or use of antihypertensive medication.

For all patients, a thorough physical examination was carried out and the New York Heart Association (NYHA) functional state was determined. HIV clinical staging was done using the WHO classification. Venous blood was taken and analysed for comprehensive blood chemistry, full blood count and CD_4_^+^ T-lymphocyte cell counts (CD_4_ cell count). Anaemia was defined as a haemoglobin level < 12 g/dl in women and < 13 g/dl in men.^14^ All tests were done at the Muhimbili National Hospital laboratory, which is their reference laboratory.

The same licensed cardiologist (PC) performed all echocardiograms on a SONOS 7500 Phillips machine with a 3.5-MHz transducer. Patients were examined in the left lateral decubitus position and the procedure followed the joint European Association of Echocardiography and American Society of Echocardiography guidelines.^15^ All tests were recorded onto magnetic optic disks and measurements were then done offline using the same Phillips echocardiogram machine.

Left ventricular (LV) mass was calculated using an autopsy-validated formula by Devereux *et al.* and indexed to body surface area to determine LV mass index (LVMI).^16^ Left ventricular hypertrophy (LVH) was considered present when LVMI was > 104 g/m^2^ in women and > 116 g/m^2^ in men.^17^

Relative wall thickness (RWT) was calculated as twice the posterior wall thickness at end-diastole divided by LV internal radius at end-diastole, and considered increased if ≥ 0.43 cm.^18^ LV end-diastolic and systolic volumes were measured using Simpson’s biplane method and were used to calculate ejection fraction, stroke volume and cardiac output as currently recommended.^15^ LV systolic dysfunction was considered present when the ejection fraction was < 50%, and diastolic dysfunction was defined as the presence of any of the following: E/A ratio < 1, mitral valve deceleration time ≥ 240 ms, or isovolumic relaxation time ≥ 105 ms.^19^

Pericardial effusion was considered present when there was an echo-free space between the visceral and parietal pericardia that persisted throughout the whole cardiac cycle. Effusion was graded as small when it was ≤ 2 cm, and large when it was > 2 cm on two-dimensional pictures during diastole.

Pulmonary hypertension was defined as echocardiographically estimated pulmonary arterial pressure > 35 mmHg with or without dilated and/or hypertrophied right ventricle and in the presence of dyspnoea.

Dilated cardiomyopathy was defined as the presence of all-chamber dilatation and global hypokinesia in the absence of features of hypertensive heart disease or any other apparent cause of global dilatation and hypokinesia. Patients were classified as having hypertensive heart disease (HHD) if they were hypertensive and found to have LVH or concentric remodelling (i.e. increase in RWT with normal LVMI), with either systolic or diastolic dysfunction, or both.

A second independent cardiologist (JL) re-read all the magnetic optical disks, and a consensus between the two cardiologists had to be reached before the final diagnosis was made.

## Statistical analysis

Data were entered and analysed using the Statistical Package for Social Sciences (SPPS) version 18. Continuous data are expressed as mean (± SD) and categorical data as number (%). Comparison between groups was done using the unpaired Student’s *t*-test for continuous variables and Chi-square test for categorical variables. Univariate and finally multivariate binary logistic regressions were performed to determine the predictors of having the different echocardiographically determined diagnoses in the HIV-positive patients. A *p*-value of less than 0.05 was considered to indicate statistical significance.

## Results

A total of 102 patients constituted the study population, 70 (68.6%) of whom were women. The patients’ mean age was 42.4 (± 11.3) years (range of 18–72). As shown in Table 1, at presentation, most patients were in the WHO HIV clinical stages 2 (42%) and 3 (42%). Five patients (4.9%) had asymptomatic HIV and 13 (12.7%) were in clinical stage 4.

**Table 1:** Socio-Demographic Characteristics And Laboratory Findings

*Characteristic*	*Men (32)*	*Women (70)*	p-*value*
Mean age (SD)	44.8 (12.6)	41.3 (10.6)	0.154
Source of referral, *n* (%)			
CTC	13 (40.6)	36 (51.4)	0.394
Wards	19 (59.4)	34 (48.6)	
Marital status, *n* (%)			
Single	10 (31.3)	15 (21.4)	0.015
Married	18 (56.3)	26 (37.1)	
Other*	4 (12.5)	29 (41.4)	
HIV duration, *n* (%)			
< 1 month	7 (21.9)	6 (8.6)	0.136
1 month – 1 year	9 (28.1)	18 (25.7)	
> 1 year	16 (50.0)	46 (65.7)	
WHO stage, *n* (%)			
Stage 1	1 (3.1)	4 (5.7)	0.457
Stage 2	10 (31.3)	32 (45.7)	
Stage 3	16 (50.0)	26 (37.1)	
Stage 4	5 (15.6)	8 (11.4)	
Smoking, *n* (%)	9 (28.1)	0 (0.0)	< 0.001
Alcohol consumption, *n* (%)	16 (50.0)	7 (10.0)	< 0.001
Taking illegal drugs, *n* (%)	3 (9.4)	0 (0.0)	0.029
Patients on HAART, *n* (%)	20 (62.5)	50 (71.4)	0.370
Mean (SD) BMI (kg/m^2^)	22.7 (3.6)	25.3 (5.4)	0.013
Mean (SD) pulse rate (beats/min)	97 (19)	94 (16)	0.404
Mean (SD) SBP (mmHg)	130 (23)	129 (20)	0.750
Mean (SD) DBP (mmHg)	80 (19)	81 (14)	0.881
Hypertension, *n* (%)	16 (50.0)	29 (41.4)	0.520
Mean (SD) RBG (mmol/l)	5.00 (1.08)	5.15 (1.37)	0.613
Diabetes, *n* (%)	2 (6.3)	4 (5.7)	0.915
Mean (SD) Hb (g/dl)	11.29 (3.44)	10.13 (3.5)	0.126
Patients with anaemia *n* (%)	27 (84)	48 (69)	0.146
Mean (SD) WBC (× 10^9^ cells/l)	5.69 (2.60)	5.62 (3.67)	0.920
Mean (SD) platelets (× 10^3^ cells/μl)	282 (129)	311 (131)	0.297
Mean (SD) ESR (mm/h)	69 (29)	71 (45)	0.823
Mean (SD) creatinine (μmol/l)	241 (407)	166 (250)	0.256
Mean (SD) cholesterol, (mmol/l)	4.55 (1.91)	4.67 (1.55)	0.729
Mean (SD) CD_4_ count (cells/μl)	203 (140)	341 (269)	0.007
Proportion with CD_4_ < 200, *n* (%)	16 (50.0)	29 (41.4)	0.520

CTC = outpatient care and treatment centre, RGB = random blood glucose, Hb = haemoglobin, WBC = white blood cell count, ESR = erythrocyte sedimentation rate *Other: cohabiting, separated, divorced, widowed.

The overall mean BMI was 24.5 (± 6.06) kg/m^2^, while the mean systolic and diastolic blood pressures were 129 and 81 mmHg, respectively. Anaemia was present in 73.5% of the total population. Forty-five (44.1%) patients had hypertension. Compared to the men, the women had a higher mean BMI (25.3 vs 22.7 kg/m^2^, *p* = 0.013) and higher CD_4_ counts (341 vs 203 cells/μl, *p* = 0.007) at the time of recruitment. On the other hand, significantly more men were smokers, consuming alcohol and taking illicit drugs (Table 1).

The most common presenting cardiac symptom was palpitations (91%), while the least common was chest pain (28%). Shortness of breath, orthopnoea, paroxysmal nocturnal dyspnoea, oedema of the lower limbs and cough were present in 69, 31, 20, 40 and 46% of the patients, respectively. Multiple complaints were common and excluding palpitations, 63% of the patients had two or more symptoms, while 37% had three or more symptoms. Thirty-two per cent of the patients were in NYHA class 4, 29% in class 3 and the rest (39%) were in class 2. Neither the presenting symptoms nor the NYHA class differed significantly between men and women.

The most common echocardiographic diagnosis was pericardial effusion, present in 41.2% of the study participants. Among these, six (5.9% of total) had symptomatic large effusions, while 36 (35.3% of total) had small effusions that were haemodynamically insignificant. Pulmonary hypertension (PHT) and dilated cardiomyopathy (DCM) were present in 12.7 and 9.8% of the participants, respectively. Hypertensive heart disease (HHD) was present in 34.3% of the participants, while mitral valve prolapse (MVP) was the diagnosis in 5.9% (Table 2).

**Table 2:** Demographic And Clinical Characteristics Of Echocardiographic Diagnoses (Univariate Analysis Comparing Patients With Echocardiographic Diagnosis And Those Without)

	*Small effusion (n = 36)*	*Large effusion (n = 6)*	*HHD (n = 35)*	*PHT (n = 13)*	*DCM (n = 10)*	*MVP (n = 6)*	*Normal (n = 18)*
Mean (SD) age (years)	39.2 (9.6)*	33.7 (8.1)*	50.6 (10.4)**	42.0 (9.1)	35.0 (8.2)*	36.6 (9.2)	40.2 (9.3)
Males (%)	31	50	40	46	20	13	17
Mean (SD) BMI (kg/m^2^)	24.5 (4.3)	24.2 (4.3)	26.7 (5.0)*	24.7 (2.9)	22.3 (5.0)	18.5 (2.7)**	23.7 (5.0)
Mean (SD) SBP (mmHg)	128 (20)	128 (20)	146 (18)**	118 (20)*	116 (16)*	115 (14)	122 (10.0)
Mean (SD) DBP (mmHg)	81 (12)	73 (12)	90 (12)**	71 (18)*	67 (22)*	75 (10)	78 (8.7)
Mean (SD) pulse rate (b/min)	103 (20)*	96 (5)	87 (13)*	96 (12)	105 (7)	90 (17)	94 (16)
Mean (SD) Hb (g/dl)	9.0 (3.1)*	8.9 (2.3)	11.8 (3.8)*	10.1 (2.0)	9.8 (1.7)	9.8 (3.4)	10.7 (3.5)
Mean (SD) WBC count in cells × 10^9^/l	6.9 (4.6)*	6.88 (3.38)	5.0 (3.1)	4.9 (1.9)	6.6 (4.5)	4.9 (1.2)	5.6 (5.6)
Mean (SD) serum creatinine (μmol/l)	328 (526)*	87 (17)	295 (503)*	204 (336)	115 (36)	158 (131)	171 (301)
Mean (SD) serum cholesterol (mmol/l)	3.9 (1.5)*	3.4 (0.7)	5.3 (1.8)*	3.9 (1.3)	3.7 (1.2)	4.7 (1.4)	5.2 (1.4)
Mean (SD) CD^4^ cell count (cells/μl)	162 (170)**	304 (283)	321 (214)	242 (208)	82 (57)*	394 (241)	449 (295)*
% on HAART	72	50	63	46	70	62	72
Mean (SD) duration on HAART (months)	24 (26)	17 (17)	41 (32)*	17 (18)	24 (24)	39 (25)	27 (20)
Mean (SD) duration after HIV diagnosis (months)	23.9 (28.9)	12 (12.4)	41.3 (40.4)^§^	18.6 (19.6)	17.0 (19.0)	34.5 (24.4)	36.2 (56.9)
Presenting cardiac symptoms (%)							
Palpitations	89	67	94	92	80	100	89
SOB	83*	100	60	95	100*	67	50
Orthopnoea	33	83	37	23	100**	33	0**
PND	19	67	11	15	90**	17	6
Oedema	53	83	40	23	100**	17	11*
Cough	61*	67	29	46	100**	33	28
Chest pain	31	33	20	31	40	50	22

SOB = shortness of breath, PND = paroxysmal nocturnal dyspnoea, HHD = hypertensive heart disease, PHT = pulmonary hypertension, DCM = dilated cardiomyopathy, MVP = mitral valve prolapse. *p < 0.05, **p < 0.01 ^§^*p* = 0.064. Note: multiple diagnoses were present.

Six (5.9%) patients had ‘other’ diagnoses, among whom two (both males, aged 47 and 31 years) had a markedly dilated aortic root and ascending aorta. The remaining four were a 33-year-old male with segmental wall motion abnormality, a 33-year-old female with rheumatic mitral valve disease, a 25-year-old female with post-partum cardiomyopathy, and a 29-year-old female with congenital valvular pulmonary stenosis and a ventricular septal defect. Eighteen (17.6%) patients had normal echocardiographic findings.

As shown in Table 2, the echocardiographic diagnoses did not differ significantly between men and women, or between patients on HAART and those not on HAART. Following a univariate analysis, it was found that compared to those without the condition, patients with small pericardial effusions were more likely to be young (39 vs 44 years, *p* = 0.033), with a higher resting pulse rate (103 vs 90 beats/min, *p* < 0.001), lower haemoglobin level (9.0 vs 11.3 g/dl, *p* = 0.002) and higher white blood cell (WBC) count (6.9 vs 4.9 × 10^9^ cells/l, *p* = 0.005). They also had higher serum creatinine levels (328 vs 129 μmol/l, *p* = 0.006), lower cholesterol levels (3.9 vs 5.0 mmol/l, *p* = 0.001) and lower CD_4_ cell counts (162 vs 373 cells/μl, *p* < 0.001).

Patients with HHD were older (51 vs 38 years, *p* < 0.001), with a higher BMI (26 vs 23 kg/m^2^, *p* = 0.003), higher systolic (148 vs 119 mmHg, *p* < 0.001) and diastolic (92 vs 74 mmHg, *p* < 0.001) blood pressure and lower mean resting pulse rate (87 vs 99 beats/min, *p* ≤ 0.001). They also had higher haemoglobin levels (12.1 vs 9.6 g/dl, *p* < 0.001), higher serum creatinine (285 vs 139 μmol/l, *p* = 0.022) and serum cholesterol levels (5.4 vs 4.2 mmol/l, *p* < 0.001), and higher mean duration on HAART (41 vs 25 months, *p* = 0.020) (Table 2).

Patients with DCM were more likely to be young (35 vs 43 years, *p* = 0.029), with lower mean systolic (116 vs 130 mmHg, *p* = 0.047) and diastolic (67 vs 82 mmHg, *p* = 0.005) blood pressures and lower CD_4_ cell counts (82 vs 322 cells/μl, *p* = 0.003). They also presented with multiple cardiac symptoms (Table 2).

Patients with MVP differed from the rest in that they had lower mean BMI (18 vs 25 kg/m^2^, *p* = 0.002) while the patients with normal echocardiographic findings had significantly higher mean CD_4_ counts (449 vs 266 cells/μl, *p* = 0.003) and were less likely to present with orthopnoea (0 vs 39%, *p* = 0.001) or oedema of the lower limbs (11 vs 48%, *p* = 0.004) (Table 2).

Echocardiographic indices differed significantly in the different diagnoses (Table 3). Of note, patients with dilated cardiomyopathy had the lowest ejection fraction while those with large pericardial effusions had the lowest stroke index. As expected, patients with hypertensive heart disease had significantly higher values of LVMI and RWT compared to patients with normal echocardiographic findings (Table 3). Among patients diagnosed with pulmonary hypertension, the mean (± SD) estimated systolic pulmonary pressure was 53 (21) mmHg.

**Table 3:** Echocardiographic Indices In The Different Diagnoses

*Variable*	*Normal (n = 18)*	*Small effusion (n = 36)*	*Large effusion (n =6)*	*HHD (n = 35)*	*PHT (n = 13)*	*DCM (n = 10)*
RV end-diastolic diameter (cm)	2.6 ± 0.5	3.2 ± 0.7*	2.8 ± 0.1	3.1 ± 0.5*	3.3 ± 0.6*	3.9 ± 0.8**
IVSd (cm)	1.02 ± 0.19	1.08 ± 0.26	0.94 ± 0.14	1.35 ± 0.27**	1.16 ± 0.25	0.83 ± 0.12*
PWTd (cm)	0.94 ± 0.16	1.04 ± 0.26	0.86 ± 0.18	1.20 ± 0.18**	1.03 ± 0.19	0.87 ± 0.18
LVIDd (cm)	4.19 ± 0.65	5.09 ± 0.98*	4.34 ± 0.75	4.77 ± 0.98	4.82 ± 0.97	6.39 ± 0.60**
RWT (cm)	0.41 ± 0.12	0.43 ± 0.15	0.41 ± 0.15	0.53 ± 0.14*	0.45 ± 0.13	0.27 ± 0.06**
LV mass index (g/m^2^)	81.9 ± 20.7	133.9 ± 56.9*	76.2 ± 16	145.7 ± 52.6**	119 ± 43*	148 ± 34**
E/A ratio	1.2 ± 0.3	1.3 ± 0.6	1.5 ± 0.5	0.97 ± 0.45	1.2 ± 0.5	1.7 ± 0.6*
MV deceleration time (ms)	192 ± 67	160 ± 62	170 ± 64	189 ± 53	182 ± 63	103 ± 48**
Isovolumic relaxation time (ms)	72 ± 24	63 ± 18	40 ± 11*	85 ± 22	73 ± 16	54 ± 12
Ejection fraction (%)	63 ± 6	49 ± 16*	65 ± 3	58 ± 15	55 ± 12	30 ± 8**
Ejection fraction < 50%, *n* (%)	0 (0)	17 (47)*	0 (0)	11 (31)	4 (31)	10 (100)**
Stroke volume (ml)	62 ± 24	66 ± 25	48 ± 25	85 ± 25*	60 ± 12	51 ± 18
Stroke index (ml/m^2^)	38.8 ± 15.7	42.6 ± 16.9	30.8 ± 17.2	50.3 ± 13.3*	36.4 ± 7.6	33.2 ± 11.5
Cardiac output (l/min)	5.8 ± 2.5	6.1 ± 2.4	4.8 ± 2.5	7.4 ± 2.5	5.7 ± 1.3	5.4 ± 2.2

RV = right ventricle, IVSd = interventricular septum in diastole, PWTd = posterior wall thickness in diastole, LVIDd = left ventricular internal diameter in diastole, RWT = relative wall thickness, LV = left ventricular, MV = mitral valve, HHD = hypertensive heart disease, PHT = pulmonary hypertension, DCM = dilated cardiomyopathy. *p < 0.05 vs normal, **p < 0.001 vs normal.

## Predictors of echocardiographic diagnoses

*Pericardial effusion*: in a model of age, gender, HIV duration, CD_4_ cell count, pulse rate, haemoglobin level and WBC count, younger age was independently associated with a diagnosis of having a large pericardial effusion (OR 0.890, 95% CI: 0.792–0.999, *p* = 0.049) (Table 4). Independent predictors of a small pericardial effusion were higher resting pulse rate (OR 1.051, 95% CI: 1.013–1.090, *p* = 0.009), low CD_4_ cell count (OR 0.996, 95% CI: 0.993–0.999, *p* = 0.004) and high WBC count (OR 1.280, 95% CI: 1.044–1.570, *p* = 0.018) (Table 4).

**Table 4:** Predictors Of Echocardiographic Diagnoses In Hiv-Infected Patients Presenting With Cardiac Symptoms

	*Univariate*		*Multivariate*	
*Echo diagnosis*	*OR (95% CI)*	*p-value*	*OR (95% CI)*	p-*value*
Small effusion				
Age (years)	0.958 (0.921–0.998)	0.037	NS	
Pulse rate (beats/min)	1.051 (1.022–1.082)	0.001	1.051 (1.013–1.090)	0.009
Hb (g/dl)	0.807 (0.701–0.930)	0.003	NS	
Cholesterol (mmol/l)	0.604 (0.436–0.837)	0.002	NS	
Creatinine (μmol/l)	1.002 (1.000–1.004)	0.027	1.002 (1.000–1.004)	0.066
WBC (× 10^9^/l)	1.210 (1.041–1.406)	0.013	1.276 (1.042–1.562)	0.018
CD_4_ (cells/μl)	0.995 (0.992–0.997)	< 0.001	0.996 (0.993–0.999)	0.004
HIV duration (months)	0.990 (0.976–1.004)	0.156	NS	
Duration HAART (months)	0.986 (0.966–1.006)	0.166	NS	
Large effusion				
Age (years)	0.907 (0.820–1.003)	0.057	0.890 (0.792–0.999)	0.049
Gender (men vs women)	0.433 (0.082–2.273)	0.322		
SBP (mmHg)	0.968 (0.927–1.010)	0.134		
Cholesterol (mmol/l)	0.461 (0.207–1.028)	0.058		
HIV duration (months)	0.959 (0.904–1.017)	0.159		
Hypertensive heart disease				
Age (years)	1.174 (1.101–1.251)	< 0.001	1.199 (1.106–1.300)	< 0.001
Gender (men vs women)	0.486 (0.205–1.150)	0.101	NS	
Use of HAART (yes/no)	1.697 (0.717–4.017)	0.229	NS	
Duration of HAART (months)	1.023 (1.002–1.044)	0.029	NS	
BMI (kg/m^2^)	1.123 (1.029–1.226)	0.010	NS	
Pulse rate (beats/min)	0.946 (0.914–0.978)	0.001	NS	
Hb (g/dl)	1.219 (1.064–1.396)	0.004	1.321 (1.052–1.658)	0.017
Cholesterol (mmol/l)	1.626 (1.223–2.163)	0.001	NS	
Creatinine (μmol/l)	1.002 (1.000–1.003)	0.046	1.004 (1.002–1.007)	0.002
HIV duration (months)	1.010 (0.998–1.021)	0.092	NS	
Pulmonary hypertension				
HIV duration (months)	0.981 (0.954–1.009)	0.190	NS	
SBP (mmHg)	0.969 (0.939–1.000)	0.050	NS	
DBP (mmHg)	0.962 (0.928–0.996)	0.030	NS	
Cholesterol (mmol/l)	0.676 (0.430–1.062)	0.089	NS	
Dilated cardiomyopathy				
Age (years)	0.921 (0.853–0.994)	0.034	NS	
BMI (kg/m^2^)	0.899 (0.777–1.040)	0.154	NS	
Pulse rate (beats/min)	1.033 (0.998–1.069)	0.062	NS	
SBP (mmHg)	0.965 (0.932–1.000)	0.050	NS	
DBP (mmHg)	0.951 (0.914–0.990)	0.013	NS	
Cholesterol (mmol/l)	0.594 (0.342–1.033)	0.065	NS	
CD_4_ (cells/μl)	0.988 (0.980–0.997)	0.010	0.988 (0.978–0.998)	0.021
HIV duration (months)	0.977 (0.944–1.012)	0.195	NS	

*Hypertensive heart disease*: increasing age was a strong and independent predictor of HHD. The odds of having HHD increased by 20% for each additional year of age (OR 1.199, 95% CI: 1.106–1.300, *p* < 0.001). Other independent predictors were higher haemoglobin level (OR 1.321, 95% CI: 1.052–1.658, *p* = 0.017) and higher serum creatinine level (OR 1.004, 95% CI: 1.002–1.007, *p* = 0.002) (Table 4). Other variables in the model were duration of HIV infection, BMI and use of HAART.

*Pulmonary hypertension*: SBP, DBP, duration of HIV infection, cholesterol level, age, gender and use of HAART were entered into the multivariate analysis. There was no single independent predictor of pulmonary hypertension.

*Dilated cardiomyopathy*: low CD_4_ cell count was an independent predictor of having an echocardiographic diagnosis of DCM (OR 0.988, 95% CI: 0.978–0.998, *p* = 0.021) after adjusting for age, gender, use of HAART and duration of HIV infection.

Adding source of referral (CTC vs wards) in the multivariate models did not alter the final results, although in univariate analyses, patients with small pericardial effusions were more likely to have been from the wards, while patients with a diagnosis of HHD were more likely to have come from the care and treatment centre (results not shown). Alcohol consumption and cigarette smoking did not show any independent association with the different diagnoses.

## Discussion

Our study documents the frequency of occurrence of echocardiographically diagnosed cardiac abnormalities of any cause in HIV-infected patients who had already presented with cardiac symptoms. We found pericardial effusion to be the main echocardiographic diagnosis among these patients. Characteristically, the effusion was small surrounding the heart and with no echocardiographically determined haemodynamic significance. Patients with small pericardial effusions were also generally sick, as evidenced by the presence of tachycardia, high WBC count, high serum creatinine levels and low CD_4_ cell count.

The finding of pericardial effusion among 41% of our patients was higher than previously reported by other investigators. Heidenreich *et al.* found the annual incidence of pericardial effusion among HIV-infected patients to be 11%.^20^ In their study they found the majority of the effusions to be small, asymptomatic and occurring more often in patients with AIDS. Since our patients were already symptomatic with cardiac complaints, this could have resulted in the higher prevalence in our study.

Heart failure from any cause can result in pericardial effusion. Since many of our patients presented with heart failure (61%), some of the pericardial effusion may have been due in part to the heart failure *per se*.

Small and large effusions are often different in aetiology. Small pericardial effusion around the heart is usually part of the effusive process that involves the pleura and the peritoneum, also known as capillary leak syndrome.^2^, ^21^ The syndrome is probably related to enhanced cytokine expression (e.g. TNF-alfa) in the later stages of HIV infection. On the other hand, large pericardial effusions in HIV disease may be related to opportunistic infections or to malignancy. Most often a clear aetiology is difficult to establish, although several studies from Africa have reported *Mycobacterium tuberculosis* as the main cause.^11^,^22^

In the multi-centre Investigation of the Management of Pericarditis In Africa (IMPI Africa) registry, microbiological evidence of tuberculosis was obtained in only 7% of the total 185 patients who were suspected of having tuberculous pericarditis,^23^ further showing the difficulties in establishing the aetiology of pericardial effusion. Other important causes of large pericardial effusions include pyogenic infection, lymphomas and Kaposi’s sarcoma.^24^,^25^

For the seven-month data-collection period, we found only six patients with large pericardial effusions. This is low when compared to 28 similar cases that were collected in a period of 18 months in 1989 in our own hospital.^11^ It is possible that the prevalence of large pericardial effusions has reduced with the use of HAART, as has been previously reported by other investigators.^26^

Second most common disease at presentation was hypertensive heart disease. Several reports have suggested that HIV patients are at a higher risk of becoming hypertensive than the general population.^27^ We found hypertensive heart disease in 34% of the patients. Interestingly, these patients had a longer duration of HIV infection and had used HAART for a longer time. Both HIV infection and use of HAART have been implicated as predisposing factors for hypertension.^7^,^8^ The mechanisms of development of hypertension in HIV-infected patients may include vasculitis in small, medium and large vessels in the form of leukocytoclastic vasculitis, and aneurysm of large vessels such as the carotid, femoral and abdominal aorta, causing impairment of flow to the renal arteries.^27^

The use of HAART has also been associated with the development of insulin resistance and other metabolic abnormalities.^28^ This could be one of the explanations for the presence of HHD in our study. However, because increasing age was independently associated with the outcome of HHD in our patients, it is possible that the usual cardiovascular risk factors had a role to play, as has been reported by other investigators.^29^,^30^

In this study, higher haemoglobin level was independently associated with the diagnosis of HHD. This was an unexpected finding, but the explanation could be that HIV-infected patients with HHD were generally less sick compared to the rest of the study patients without HHD and therefore had significantly better haemoglobin levels. It should be emphasised however that the average haemoglobin level in the patients with HHD (11.8 g/dl) was slightly lower than normal.

The incidence of HIV-associated pulmonary hypertension is estimated to be 1/200; much higher than the 1/200 000 found in the general population.^31^ In our cohort, pulmonary hypertension was present in 13% of the patients. Niakara *et al.* found the prevalence of pulmonary hypertension to be 5% in HIV-infected patients hospitalised in a cardiac unit in Harare.^32^ The difference between our study and that of Niakara could be that his study was retrospective and some cases of pulmonary hypertension could have been missed because no clear definition was set beforehand. Our cohort included out-patients, therefore increasing the chances of having more cases with pulmonary hypertension.

The usual presentation of patients with pulmonary hypertension is shortness of breath, and in most cases this is attributed to lung disease. These patients generally appear to have no advanced immunosuppression, in other words no relation to the disease stage as reflected by CD_4_ count.^33^ The majority of our patients presented with shortness of breath, which was out of proportion to the physical findings, and the average CD_4_ count was 242 ± 208 cells/μl. This is similar to that reported by Le Houssine *et al.*^34^ in their analysis of nine HIV-infected patients with primary pulmonary hypertension, in which the mean CD_4_ count was 234 ± 217cells/μl.

**Fig. 1:**
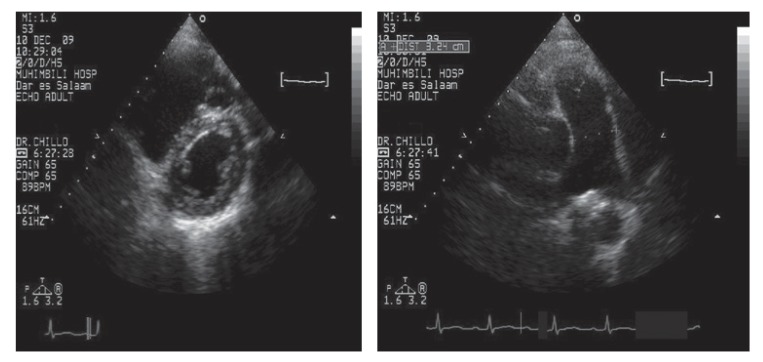
D-sign (left image) and dilated main pulmonary artery (right image) in a patient with pulmonary hypertension.

In multivariate analysis, we found no single predictor of pulmonary hypertension. The echocardiographic findings were those of dilated right heart with a prominent D-sign on parasternal short-axis view. Often the main pulmonary artery was also dilated (Fig. 1). The prognosis of these patients is generally poor, with median survival of approximately six months.^33^

Dilated cardiomyopathy is probably the most studied form of heart muscle disease in HIV-infected patients. The prevalence of dilated cardiomyopathy has been reported to be up to 40%, with an annual rate of incidence of 15.9 cases per 1 000 patients.^35^ The condition is generally associated with advanced immunosuppression and poor outcome.^36^

We found 10 cases (9.8%) of dilated cardiomyopathy in a seven-month period. All 10 patients were in heart failure and with advanced immunosuppression, with an average CD_4_ cell count of 83 cells/μl. These patients were generally young with a mean age 35 years and short duration after HIV diagnosis. Often the first presentation to hospital was heart failure.

The formation of dilated cardiomyopathy is thought to be due to either the direct action of HIV on the myocardial tissue or to an autoimmune process induced by HIV, possibly in association with other cardiotropic viruses.^37^ Poor nutritional status has also been implicated and may be a greater role player in sub-Saharan Africa where idiopathic dilated cardiomyopathy is also prevalent.^38^ Nutritional deficiencies are common in HIV infection, particularly in late stages of the disease and may be due to poor absorption of food and prolonged diarrhoea.^39^ Deficiency of trace elements such as selenium have also been directly or indirectly associated with cardiomyopathy.^40^,^41^

The finding of two cases of aneurismal dilatation of the aorta in our study is not unique. This pathology has been reported by other investigators.^42^,^43^ These case reports describe aneurysms of the aorta and peripheral cerebrovascular arteries, sometimes necessitating surgical repair. The aneurysms are probably a result of vasculitic changes induced by the virus, or by other infectious causes such as cytomegalovirus and tuberculosis.^44^ However, an infective agent was not always identified.^44^

The inclusion in our study of patients with palpitations could have resulted in over-inclusiveness, because palpitation as a symptom is non-specific. However, most patients had palpitations plus other cardiac symptoms, since the majority of our patients had more than one symptom. In fact, palpitations was a symptom in 91% of the patients.

The majority of patients in this cohort had anaemia, which is often accompanied by palpitations. Unless heart failure has occurred, the echocardiographic findings in these patients would most likely be normal. This could explain the 18 patients in our study who had a normal echocardiograph. Shortness of breath is a symptom of lung disease and if these patients were also anaemic and had tachycardia, it would be easy to surmise they had a cardiac condition, further explaining the normal echocardiographs.

We acknowledge several limitations of this study. First, although this study was powered to detect differences in cardiac involvement between patients with different immunological status, the same was not true for other factors that may also have contributed to the development of specific cardiac conditions, such as alcohol consumption and cigarette smoking. This could have resulted in the lack of association.

Second, this study did not include viral load and socio-economic status, which are important predictors of dilated cardiomyopathy, as reported by Twagirumukiza and co-workers.^9^ Third, as is the case for all cross-sectional studies, a causal relationship cannot be claimed and therefore the findings of this study should be interpreted with caution, as it is not possible to tell whether HIV infection resulted in cardiac disease or vice versa.

## Conclusion

The pattern of cardiac abnormalities in this era of HAART is still dominated by pericardial disease and cardiomyopathy, as seen in the patients presenting with cardiac symptoms in our hospital. Hypertensive heart disease may become an important cause of cardiac involvement in the future. Large prospective studies are needed in order to confirm this observation.
